# Impact of Five Nights of Sleep Restriction on Glucose Metabolism, Leptin and Testosterone in Young Adult Men

**DOI:** 10.1371/journal.pone.0041218

**Published:** 2012-07-23

**Authors:** Amy C. Reynolds, Jillian Dorrian, Peter Y. Liu, Hans P. A. Van Dongen, Gary A. Wittert, Lee J. Harmer, Siobhan Banks

**Affiliations:** 1 Centre for Sleep Research, University of South Australia, Adelaide, South Australia, Australia; 2 Los Angeles Biomedical Research Institute at Harbor-University of Los Angeles Medical Center, Torrance, California, United States of America; 3 Sleep and Performance Research Center, Washington State University, Spokane, Washington, United States of America; 4 Discipline of Medicine, University of Adelaide, Adelaide, South Australia, Australia; 5 Adelaide Institute for Sleep Health, Repatriation General Hospital, Daw Park, South Australia, Australia; Simon Fraser University, Canada

## Abstract

**Background:**

Sleep restriction is associated with development of metabolic ill-health, and hormonal mechanisms may underlie these effects. The aim of this study was to determine the impact of short term sleep restriction on male health, particularly glucose metabolism, by examining adrenocorticotropic hormone (ACTH), cortisol, glucose, insulin, triglycerides, leptin, testosterone, and sex hormone binding globulin (SHBG).

**Methodology/Principal Findings:**

N = 14 healthy men (aged 27.4±3.8, BMI 23.5±2.9) underwent a laboratory-based sleep restriction protocol consisting of 2 baseline nights of 10 h time in bed (TIB) (B1, B2; 22:00–08:00), followed by 5 nights of 4 h TIB (SR1–SR5; 04:00–08:00) and a recovery night of 10 h TIB (R1; 22:00–08:00). Subjects were allowed to move freely inside the laboratory; no strenuous activity was permitted during the study. Food intake was controlled, with subjects consuming an average 2000 kcal/day. Blood was sampled through an indwelling catheter on B1 and SR5, at 09:00 (fasting) and then every 2 hours from 10:00–20:00. On SR5 relative to B1, glucose (*F*_1,168_ = 25.3, *p*<0.001) and insulin (*F*_1,168_ = 12.2, *p*<0.001) were increased, triglycerides (*F*_1,168_ = 7.5, *p* = 0.007) fell and there was no significant change in fasting homeostatic model assessment (HOMA) determined insulin resistance (*F*_1,168_ = 1.3, *p* = 0.18). Also, cortisol (*F*_1,168_ = 10.2, *p* = 0.002) and leptin (*F*_1,168_ = 10.7, *p* = 0.001) increased, sex hormone binding globulin (*F*_1,167_ = 12.1, *p*<0.001) fell and there were no significant changes in ACTH (*F*_1,168_ = 0.3, *p* = 0.59) or total testosterone (*F*_1,168_ = 2.8, *p* = 0.089).

**Conclusions/Significance:**

Sleep restriction impaired glucose, but improved lipid metabolism. This was associated with an increase in afternoon cortisol, without significant changes in ACTH, suggesting enhanced adrenal reactivity. Increased cortisol and reduced sex hormone binding globulin (SHBG) are both consistent with development of insulin resistance, although hepatic insulin resistance calculated from fasting HOMA did not change significantly. Short term sleep curtailment leads to changes in glucose metabolism and adrenal reactivity, which when experienced repeatedly may increase the risk for type 2 diabetes.

## Introduction

Chronic sleep restriction is common due to occupational, social and lifestyle demands and/or medical conditions [1]. In large cross-sectional and cohort studies, self-reported short sleep duration has been found to be related to metabolic disorders such as insulin resistance [2], obesity [3]–[5], metabolic syndrome [6] and type 2 diabetes [7]–[9]. Key features of such metabolic disorders include development of insulin resistance, hyperglycemia and hypertriglyceridemia. Indeed, alterations in insulin, glucose and triglycerides are indicative of metabolic syndrome [10]–[14]. Recent controlled laboratory studies have investigated the causal relationship between short sleep and metabolic health and have generally reported impaired glucose tolerance and/or development of insulin resistance after one to fourteen nights of four to six hours of sleep restriction per day [15]–[21]. Considering the broad health implications this could have in today's 24/7 society, there is considerable interest in elucidating the hormonal mechanisms underlying these sleep loss-related metabolic changes.

Sleep restriction impairs glucose tolerance [16], [18]. Early studies reported little change in circulating insulin with sleep restriction [18], [22], but subsequent studies have found that insulin increases [15], [16], [20], particularly after breakfast [16]. Overall insulin resistance has generally been found to worsen with sleep restriction [15], [23]. Little information exists on the effects of sleep restriction on triglycerides or free fatty acids, which are converted from triglycerides in many tissues. Fasting free fatty acids (FFA) show no significant change after one night of sleep restriction [15] and increase following two [16] nights of sleep restriction to 4 h TIB. Cross-sectional data suggest a relationship between short sleep duration and elevated triglycerides [24]. To date, the effect of sleep restriction on triglycerides has only been experimentally examined in women, with no significant change found following three nights of 4 h time in bed (TIB) [25].

Cortisol, a stress hormone released by the adrenal axis, induces gluconeogenesis, reduces peripheral glucose utilization, induces insulin resistance and raises blood glucose concentrations [26]. Some studies showed that sleep restriction significantly increases plasma cortisol levels during the late afternoon to early evening hours [17], [18], whereas others showed little to no changes [16], [21], [23], [27]. These conflicting results may be due to the variations in physiological stress induced by the different forms of sleep restriction (e.g. amount and timing of sleep), invasiveness of the tests used during the study (e.g. insertion of indwelling catheters, fasting, etc.) and the type of laboratory environment (e.g. amount of social contact). Effects of sleep loss on the adrenal axis, potentially as a stress response, is one hormonal mechanism by which altered sleep could cause changes in glucose metabolism, either directly acting on the adrenal gland or indirectly through changes in pituitary hormones such as adrenocorticotropic hormone (ACTH).

Leptin is an adipokine that acts as a satiety signal, and can directly or indirectly improve insulin sensitivity and reduce lipids and visceral adiposity [28]. Early studies investigating the relationship between sleep restriction and leptin showed that sleep restriction decreased leptin levels [17], [19], and increased self-reported appetite and hunger [19]. These data implicate reduced leptin as a hormonal mechanism by which sleep restriction can worsen insulin sensitivity, increase circulating lipids, and lead to obesity and abdominal obesity via disruption of energy balance and increased cravings for carbohydrate rich foods. Subsequent studies have not consistently confirmed these earlier findings [20], [29]–[34], possibly due to differences in experimental protocols, including whether subjects were kept in the laboratory for the duration of the study period or allowed to leave during the day [18], [19], [21]; ad libitum [29] versus controlled [35] versus restricted food intake [17]; use of glucose tolerance tests in place of a breakfast meal [17], [19], [23] which contributes to restricted or abnormal eating patterns over the day; or whether subjects were rested in bed during blood sampling [17], [18], [21]. Although more data are needed under each of these unique conditions to elucidate the impact sleep restriction has on leptin, it is widely considered a candidate mechanism for the development of metabolic diseases such as obesity and type 2 diabetes.

Testosterone is the quintessential anabolic hormone, vital for masculinization, and responsible for increased muscle mass and strength and reduced adiposity. Lowering testosterone has the inverse effect on metabolic and body compositional parameters, including increases of overall adiposity [36] and visceral fat accumulation [37] and induction of insulin resistance as well as decrease of resting metabolic rates. Both low testosterone [38]–[40] and sex hormone binding globulin (SHBG) [41], [42] are associated with metabolic syndrome and insulin resistance. While observational studies have identified a relationship between total sleep deprivation and reduced testosterone [43], [44], only one experimental laboratory based study has investigated the impact of partial sleep restriction on testosterone [45]. It found a decrease in afternoon and evening (14:00–22:00) plasma testosterone levels after eight nights of sleep restriction to five hours per night.

No previous studies have examined the effect of sleep restriction on SHBG, a hepatic glycoprotein primarily responsible for transport of the sex hormones testosterone and estradiol [46]. Given associations between SHBG and metabolic functioning in cohort studies [41], [42], it may be important to examine the impact of sleep restriction on SHBG. Remarkably, while most studies of sleep restriction and metabolism have exclusively examined male populations, the effects of sleep loss on the regulation of sex hormones has received scant attention.

The overall aim of this study was to address gaps in knowledge that still exist regarding sleep restriction and metabolism by examining the effect of 5 nights of sleep restriction on glucose metabolism and triglycerides, and to examine cortisol-, leptin- or testosterone-based mechanisms that may underpin these changes in healthy young men.

## Materials and Methods

### Ethics Statement

The study was approved by the University of South Australia Human Research Ethics Committee. All subjects provided written informed consent to participate in the study prior to entering the laboratory. Study subjects were paid a modest honorarium for their time.

### Subjects

Sixteen healthy men aged 22–36 y (mean ± SD; 27.4±3.8) were recruited to the study. 28.6% were from racial/ethnic minorities. Subjects reported habitual nocturnal sleep durations between 7.0 h and 8.5 h (8.2±1.3) and habitually rose between 06:00 and 09:00. Subjects did not report habitual napping (confirmed using actigraphy for a minimum 1 week prior to participating in the study), or any complaints of insomnia, daytime sleepiness, or other sleep-wake disturbances. Subjects completed interviews, clinical history, questionnaires and blood and urine tests, which confirmed that they did not present with any acute or chronic medical and psychological conditions. None of the subjects were extreme morning or evening types [47].

Subjects who participated in the study were non-smokers with a body mass index (BMI) between 19 and 30 kg/m^2^ (23.5±2.9). Subjects were included if they were not engaged in shift work, transmeridian travel or unusual sleep/wake routines in the two months prior to the study. Sleep health was confirmed with the first night of laboratory PSG and questionnaires [47], [48]. Circadian phase position was assessed with a morningness/eveningness questionnaire [47], which was completed during pre-study screening.

During the week prior to the laboratory phase of the study, all subjects were monitored at home with actigraphy and sleep-wake diaries. Subjects were instructed to avoid using caffeine, alcohol, tobacco and medications for a minimum of one week before the laboratory experiment. Compliance was verified by blood and urine screens.

Of the original 16 subjects deemed eligible for the study, one did not commence the study due to work commitments, and another elected to leave the laboratory prior to completion for personal reasons, leaving a final sample size of N = 14.

### Experimental Protocol

Subjects were studied in groups of 3 or 4 at a time for 9 consecutive days in the Centre for Sleep Research at the University of South Australia. All subjects underwent two initial nights of baseline sleep of 10 h TIB (B1, B2; 22:00–08:00), followed by 5 nights of 4 h TIB (SR1–SR5; 04:00–08:00), and a recovery night of 10 h TIB (R1; 22:00–08:00). Subjects left the laboratory mid-morning following R1. All subjects completed the same protocol. Trained research staff monitored subjects continuously during the study period to ensure compliance with the experimental protocol. Subjects also had daily contact with the Principal Investigator.

During wake periods, subjects completed a driving simulation task and neurobehavioral test batteries consisting of questionnaires and cognitive performance tests. VASs for hunger, appetite and satiety were completed daily. On B1 and SR5, these scales were completed at 11:00, 12:30, 16:30 and 19:30. Subjects rated their hunger (1 = ‘not hungry at all’ to 9 = ‘as hungry as I've ever felt’), appetite (1 = ‘very weak desire to eat’ to 9 = ‘very strong desire to eat’) and satiety (1 = ‘not at all full’ to 9 = ‘as full as I've ever felt’). The other questionnaires and cognitive performance tests will be reported elsewhere.

When not engaged in tasks, subjects were allowed to watch movies, interact with each other and with study staff, and engage in activities, such as card games and board games. While able to move freely inside the laboratory, subjects were told that vigorous exercise, or games which involved excess movement, were not permitted.

Subjects wore a wrist actigraph throughout the 9-day laboratory protocol. On B1, B2, SR2, SR3, SR5 and R1 sleep was recorded with PSG using a standard montage. On all days except those with blood draws (B1, SR5), subjects were given the opportunity to shower between 08:30 and 09:30. The light levels in the laboratory were kept at less than 20 lux during wake periods and less than 1 lux (lights off) during sleep periods. Ambient temperature was maintained between 22°C and 24°C.

### Food Consumption

Food intake was controlled during the laboratory phase of the study. Subjects chose preferred food items from a limited menu prior to entering the laboratory, but serving sizes and access to food (timing and duration) was determined by the research team to ensure consistency across days and subjects. Target energy intake was 2000 kcal per day. Subjects ate an identical meal for breakfast, lunch and dinner on SR5 to what they consumed on B1.

### Blood Sampling and Continuous Glucose Monitoring

Blood draws were conducted on B1 and SR5 by two clinical nurses. An intravenous 16G catheter was inserted at 08:15 into the cubital fossa vein, and removed at approximately 20:30 the same day. Seven blood draws occurred over the day, with approximately 15 ml collected per draw (5 ml for serum and 10 ml for plasma) using BD Vacutainer**®** blood collection tubes. The maximum volume of blood taken per day was 105 ml. Pre-prandial blood was drawn at 09:00, after which subjects consumed a set breakfast. Blood was then drawn at 10:00, 12:00, 14:00, 16:00, 18:00 and 20:00. Blood samples were immediately cold centrifuged. Serum and plasma were stored in 1.5 ml aliquots, frozen at −20°C, and batched for assaying.

The Medtronic Guardian**®** REAL-Time Continuous Glucose Monitoring System was used to monitor interstitial glucose for 24 h at baseline and after sleep restriction. The monitoring system consisted of a 5 mm, hair-like sensor, which was inserted subcutaneously 5 cm to the right of the navel, and a recording device worn on the belt. Interstitial glucose levels were recorded every 5 min. The sensor was calibrated every 2 h with capillary blood, using the Accu-check Softclix Lancing Device LD01, Optium glucose electrode test strips and Optium Xceed Blood Glucose Meter. The sensor was tolerated well by all subjects. The Medtronic Guardian® REAL-Time has been validated against plasma glucose [49] and is widely used in clinical settings [50], [51].

### Assays

Plasma glucose was measured on a Hitachi 912 Chemistry analyzer by the hexokinase method using kits from Roche Diagnostics (Castle Hill, NSW, Australia). Plasma insulin (HI-11K) and leptin (HL-81K) were assayed by radioimmunoassay (RIA) according to the manufacturer's instructions using kits obtained from Millipore (Billerica, MA, USA). The intra-assay coefficients of variation (CV) were below 10% for both assays. Serum concentrations of triglycerides were measured with the Siemens Advia 1650 Clinical Chemistry System (Siemens Healthcare Diagnostics, Regency Park, SA, Australia). Total testosterone and sex hormone binding globulin concentrations were determined in serum using the Cobas electrochemiluminescence immunoassay (Roche Diagnostics Ltd., Indianapolis, IN, USA) on the Roche Modular E170 (Roche Diagnostics Ltd, Indianapolois, IN, USA). For sex hormone binding globulin (SHBG) measurement, serum was diluted 1∶21 by adding provided SHBG diluents. The CV was 9.3% at a concentration of 10.7 nmol/l for total testosterone and 4.0% at a concentration of 32.3 nmol/l for SHBG. Serum ACTH and cortisol were assayed by RIA in a single run according to the manufacturer's instructions on the Immulite 1000 (Siemens Healthcare Diagnostics, Regency Park, SA, Australia). This included ensuring supplied quality control samples were within the acceptable ranges prior to each run.

### Statistical Analysis

Analyses were performed using SAS 9.2 (SAS Institute Inc., Cary, NC, USA). Mixed-effects ANOVAs were conducted to test effects of sleep restriction on metabolic and hormonal profiles. Condition (B1, SR5), time of day (09:00, 10:00, 12:00, 14:00, 16:00, 18:00, 20:00) and condition*time of day interaction were specified as fixed effects, and a random effect on the intercept was included. Planned contrasts were conducted for ACTH and cortisol to specifically examine the afternoon and early evening period as previous studies found elevation of these hormones during these times after sleep restriction.

## Results

Fourteen male subjects completed the study. Of the original 16 subjects deemed eligible for the study, one did not commence the study due to work commitments, and another elected to leave the laboratory prior to completion for personal reasons.

### Sleep Architecture

Polysomnography (PSG) was conducted on B1 (10 h TIB) and SR5 (4 h TIB). As expected, total sleep time (TST) was reduced on SR5 (mean ± SD: 233.6±1.1 min) compared with B1 (519.4±8.9 min, *F*_1,13_ = 1034.6, *p*<0.001). Reductions following sleep restriction were also found for sleep onset latency (B1 = 8.9±1.9 min; SR5 = 0.6±0.2 min, *F*_1,13_ = 19.2, *p*<0.001), and wake after sleep onset (B1 = 55.5±8.6 min; SR5 = 6.8±5.1 min, *F*_1,13_ = 33.3, *p*<0.001). Time spent in Stage 1 (B1 = 10.5±2.5 min; SR5 = 2.7±0.8 min, *F*_1,13_ = 15.5, *p* = 0.002), and Stage 2 (B1 = 243.0±0.4 min; SR5 = 70.0±5.3 min, *F*_1,13_ = 308.8, *p*<0.001) were likewise reduced on SR5. Time spent in slow wave sleep was reduced in SR5 (B1 = 129.2±10.8 min; SR5 = 95.6±6.7 min, *F*_1,13_ = 18.4, *p*<0.001). Minutes spent in REM sleep were also reduced following sleep restriction (B1 = 136.6±8.3 min; SR5 = 65.3±5.1 min; *F*_1,13_ = 61.9, *p*<0.001).

### Caloric Intake

Food consumption was strictly controlled throughout the study. Subjects consumed identical meals on B1 and SR5. Average caloric intake (mean ± SD) was 1973.0±31.9 kcal on B1 and 1961.8±30.9 kcal on SR5. Carbohydrates accounted for 52.9%±2.2% of caloric intake on B1 and 52.7%±2.3% on SR5; fats accounted for 26.9%±1.5% of caloric intake on B1 and 27.1%±1.7% on SR5; and proteins accounted for 16.8%±0.8% of caloric intake on B1 and 16.9±0.9% on SR5.

A summary of the results of the mixed-effects ANOVAs for all blood measures can be found in Table 1.

**Table 1:** Statistical results from mixed-effects ANOVAs for all metabolic and hormonal variables.

			Regression Output				
Variable	B1	SR5		F	DF_NUM_	DF_DENOM_	p
ACTH	21.5(1.6)	22.1(1.1)	*Condition*	0.3	1	168	.59
(pg/ml)			*Time of Day*	15.2	6	168	<.001
			Condition*Time of Day	0.3	6	168	.94
Cortisol	292.2(18.1)	337.4(16.9)	*Condition*	10.2	1	168	.002
(nmol/l)			*Time of Day*	48.6	6	168	<.001
			Condition*Time of Day	1.1	1	168	.35
Glucose	4.9(0.1)	5.5(0.1)	*Condition*	25.3	1	168	<.001
(mmol/l)			*Time of Day*	11.6	6	168	<.001
			Condition*Time of Day	4.8	6	168	<.001
Insulin	34.4(2.8)	44.2(4.2)	*Condition*	12.2	1	168	<.001
(uU/ml)			*Time of Day*	37.4	6	168	<.001
			Condition*Time of Day	3.9	6	168	.001
Triglycerides	1.5(0.1)	1.4(0.1)	*Condition*	7.5	1	168	.007
(mmol/l)			*Time of Day*	12.7	6	168	<.001
			Condition*Time of Day	0.2	6	168	.97
Leptin	4.3(0.3)	4.8(0.4)	*Condition*	10.7	1	168	.001
(ng/ml)			*Time of Day*	9.8	6	168	<.001
			Condition*Time of Day	0.5	6	168	.80
Testosterone	14.7(0.5)	14.0(0.5)	*Condition*	2.8	1	168	.09
(nmol/l)			*Time of Day*	11.2	6	168	<.001
			Condition*Time of Day	0.5	6	168	.85
SHBG	31.4(1.2)	27.9(1.0)	*Condition*	12.1	1	168	<.001
(nmol/l)			*Time of Day*	1.4	6	168	.21
			Condition*Time of Day	0.4	6	168	.86
HOMA	8.3(0.8)	12.1(1.3)	*Condition*	19.3	1	168	<.001
			*Time of Day*	35.1	6	168	<.001
			Condition*Time of Day	5.4	6	168	<.001

Note: Results from the Linear Mixed Models Analysis are shown including the mean (± SE) values from B1 and SR5, *F* value, numerator degrees of freedom (DF_NUM_), denominator degrees of freedom (DF_DENOM_) and significance (*p*) values. ACTH, adrenocorticotropic hormone; SHBG, sex hormone binding globulin; HOMA, homeostatic model assessment. ‘Condition’ refers to a difference between B1 and SR5, while ‘Time of Day’ refers to the change over the day of blood sampling. There was a significant day effect (B1 compared with SR5) for cortisol, glucose, insulin, triglycerides, leptin and sex hormone binding globulin. A trend for decreasing testosterone was found. A significant draw effect (change in levels within B1 and SR5) was found for ACTH, cortisol, glucose, insulin, triglycerides, leptin and testosterone. Day by draw interactions were found for glucose and insulin. pg/ml, picogram per milliliter; nmol/l, nanomol per liter; mmol/l, millimol per liter; uU/ml, micromoles per milliliter; and ng/ml, nanograms per milliliter.

### Adrenal Axis - Adrenocorticotropic Hormone (ACTH) and Cortisol

**Figure 1:**
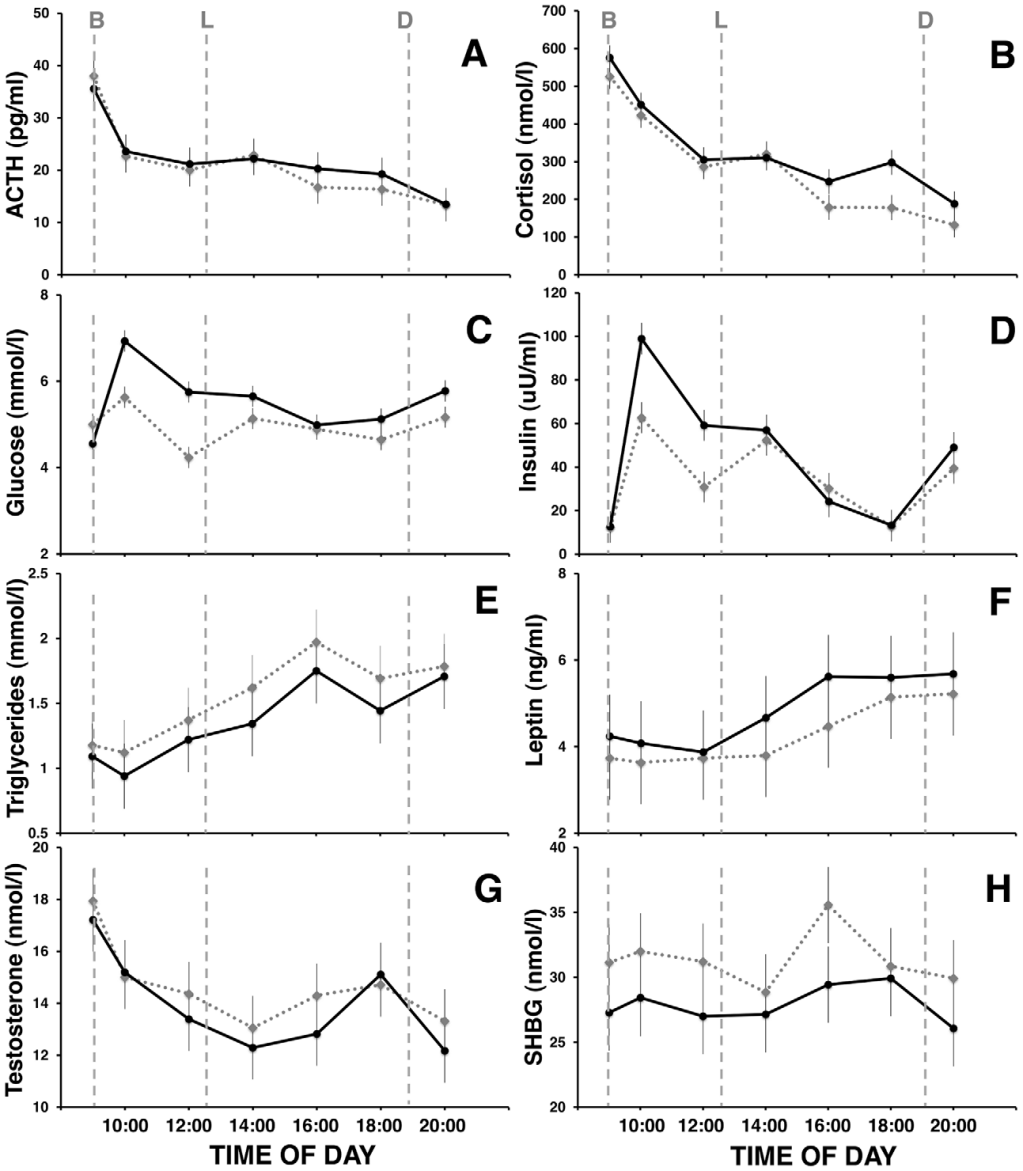
Metabolic and hormonal outcomes on baseline day 1 (B1) and after 5 nights of sleep restriction (SR5). Mean levels of: **A**–ACTH, **B**–cortisol, **C**–glucose, **D**–insulin, **E**–triglycerides, **F**–leptin, **G**–total testosterone, and **H**–sex hormone binding globulin. Bars represent SE. All subjects experienced two baseline nights of 10 h TIB first; the dashed grey curve shows data from the first baseline day (B1). Subjects then underwent 5 nights of sleep restriction to 4 h TIB; the solid black curve shows data from the last sleep-restricted day (SR5). On each of these two days, blood was drawn at 09:00 (fasting) and then every 2 h from 10:00 to 20:00. Vertical dashed grey lines represent meal opportunities; B, breakfast; L, lunch; D, dinner.

Mean ACTH (Figure 1A) decreased across time of day on both B1 and SR5, demonstrating a normal diurnal pattern (*p*<0.001). ACTH was 3.2% higher after sleep restriction (mean ± SE 22.1±1.1 pg/ml) than at baseline (21.5±1.6 pg/ml), but this increase was not statistically significant (*p* = 0.59). No condition*time of day interaction was found (*p* = 0.94). These results are summarized in Table 1. Previous studies have shown largest differences between baseline and sleep restricted levels of both ACTH and cortisol to be in the late afternoon. The greatest difference between B1 and SR5 ACTH in the present data was observed between 16:00 and 20:00. Planned contrasts were conducted on ACTH and cortisol (results for cortisol reported below) as previous studies have shown that sleep restriction prevents the usual afternoon and evening drop in these hormones. The analysis revealed that ACTH levels were higher at 16:00 on SR5 (mean ± SEM; 20.2±1.4) than B1 (16.8±1.5; *F*_1,97_ = 4.7, *p* = 0.03), but not at 18:00 (SR5: 19.3±2.3, B1: 16.4±1.4; *F*_1,97_ = 1.5, *p* = 0.22) or 20:00 (SR5: 13.5±1.0, B1: 13.4±1.0; *F*_1,97_ = 0.02, *p* = 0.88).

Mean cortisol (Figure 1B) decreased across time of day on both B1 and SR5, reflecting the normal diurnal rhythm in cortisol (*p*<.001). Cortisol was 15.5% higher after sleep restriction (mean ± SE: 337.4±16.9 nmol/L) than at baseline (292.2±18.1), and this increase was statistically significant (*p* = 0.002). No condition*time of day interaction was found (*p* = .35). There was an increase in cortisol between 16:00 and 20:00 following sleep restriction compared with baseline. Planned contrasts showed that cortisol levels were significantly higher at 16:00 on SR5 (mean ± SEM; 247.1±21.9) than B1 (179.0±16.6; *F*_1,97_ = 5.5, *p* = 0.02) and at 18:00 (SR5: 298.0±41.4, B1: 178.1±17.9; *F*_1,97_ = 13.3, *p*<0.001). No significant difference was found between SR5 (188.3±25.9) and B1 (132.2±13.2) at 20:00 (*F*_1,97_ = 2.9, *p* = 0.09).

### Glucose Metabolism - Glucose, Insulin and Triglycerides

**Figure 2:**
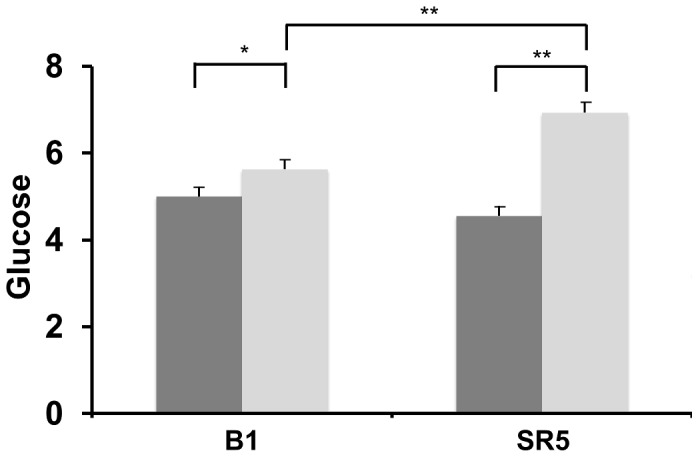
Blood glucose levels before and after breakfast on baseline day 1 (B1) and after 5 nights of sleep restriction (SR5). Mean pre-prandial (dark grey) and post- prandial (light grey) blood glucose (mmol/l) at baseline (B1) and following sleep restriction (SR5) (*, *p* = .002, **, *p*<.001) (bars = SE).

Mean blood glucose (Figure 1C) decreased across time of day on both B1 and SR5, reflecting the normal diurnal rhythm in glucose (*p*<0.001). Glucose was 11.4% higher after sleep restriction (mean ± SE; 5.5±0.1) than at baseline (4.9±0.1), and this increase was statistically significant (*p*<0.001). A condition*time of day interaction was also found, which was indicative of an amplified response to meals after sleep restriction (*p*<.001). Figure 2 shows breakfast pre-prandial (09:00) and post-prandial (10:00) plasma glucose at B1 and SR5. There was a difference between pre- and post-prandial glucose on B1 (*p*<.001), and between pre- and post-prandial glucose on SR5 (*p*<.001). There was no difference between pre-prandial glucose on B1 and SR5 (*p* = 0.13), but there was between B1 and SR5 post-prandial glucose (*p*<.001), suggesting an impaired response to ingestion of the breakfast meal following sleep restriction (see Figure 2). Fasting homeostatic model assessment (HOMA) did not change significantly on SR5 (mean ± SE; 2.9±1.2) compared with B1 (2.6±1.1, *p* = 0.18). However, a difference was found between B1 and SR5 HOMA across the day (*p*<0.001), with time of day (*p*<0.001) and condition*time of day effects also found (*p*<0.001).

**Figure 3:**
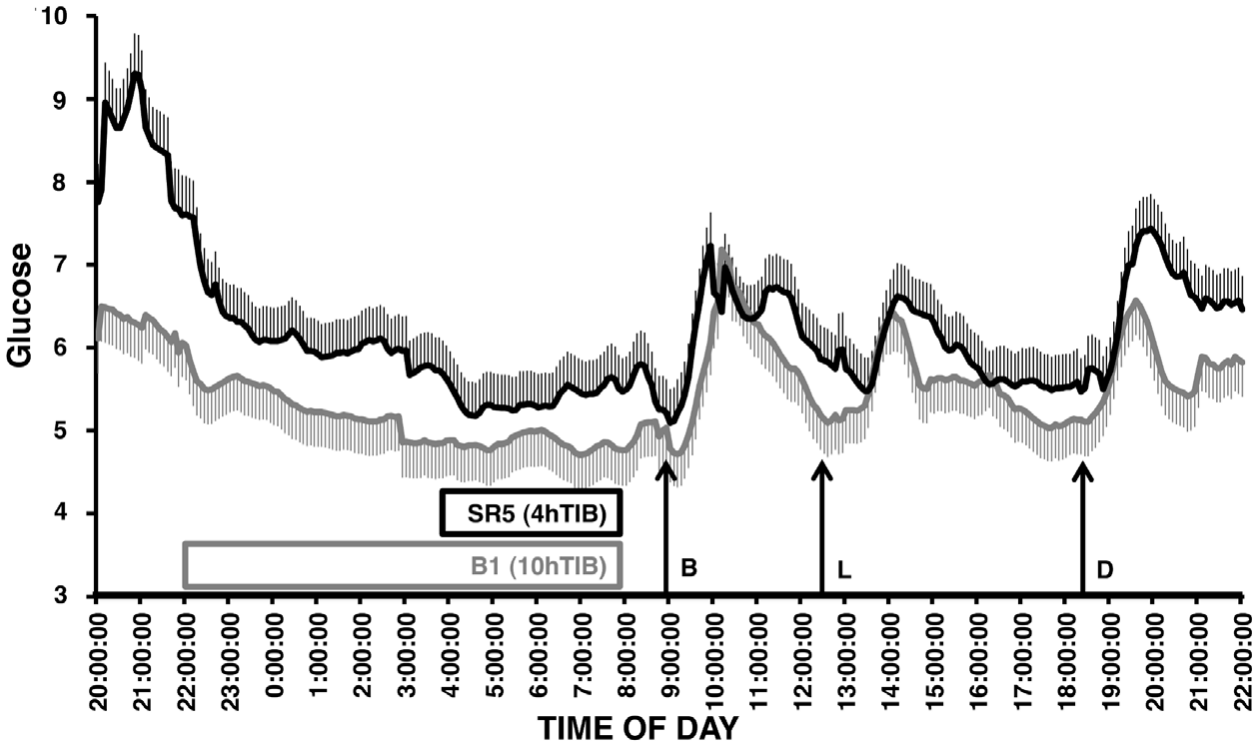
24 h interstitial glucose (mmol/l) on baseline day 1 (B1) and after 5 nights of sleep restriction (SR5). Readings were taken at 5 min intervals starting at 20:00 using the Guardian**®** REAL-Time Continuous Glucose Monitoring System. Meal times are marked with arrows, left to right: breakfast (B) (09:00), lunch (L) (13:00), and dinner (D) (18:30). Baseline day 1 interstitial glucose is shown in grey; sleep restriction day 5 is shown in black (bars = SE).

Elevated glucose was also seen in interstitial fluid (Figure 3). Interstitial glucose after sleep restriction was elevated compared with baseline (*F*_1,7596_ = 618.4, p<0.001), and a significant time of day effect was also found (*F*_312,7596_ = 6.0, *p*<0.001). No condition*time of day interaction was found (*F*_312,7596_ = 1.1, *p* = 0.20).

Mean insulin (Figure 1D) decreased across time of day on both B1 and SR5 (*p*<0.001). Insulin was 28.8% higher after sleep restriction (mean ± SE: 44.2±4.2 uU/ml) than at baseline (34.4±2.8), and this increase was statistically significant (*p*<0.001). A condition*time of day interaction was also found, and appeared to be indicative of an amplified response to meals after sleep restriction (*p* = 0.001).

Mean triglycerides (Figure 1E) increased across time of day on both B1 and SR5, peaking at 16:00, and reflecting significant change in levels across the day (*p*<0.001). Triglycerides were 11.0% lower after sleep restriction (mean ± SE: 1.37±0.1 mmol/l) than at baseline (1.53±0.0), and this decrease was statistically significant (*p* = 0.007). No significant condition*time of day interaction effect was found (*p* = 0.97).

### Leptin

Mean leptin (Figure 1F) increased across time of day on both B1 and SR5, reflecting normal rhythm (*p*<0.001). Leptin was 13.7% higher after sleep restriction (mean ± SE: 4.8±0.4 ng/ml) than at baseline (4.3±0.3), and this increase was significant (*p* = 0.001). No condition*time of day interaction effect was found (*p* = 0.80).

### Testosterone and Sex Hormone Binding Globulin (SHBG)

Total testosterone (Figure 1G) decreased across time of day on both B1 and SR5, reflecting testosterone's normal diurnal rhythm (*p*<0.001). No significant effect of condition (*p* = 0.09), or condition*time of day interaction (*p* = 0.85) was found.

Sex hormone binding globulin (SHBG; Figure 1H) did not show any time of day effects (*p* = 0.21). SHBG was 11.0% lower after sleep restriction (mean ± SE: 27.9±1.0 nmol/L) than at baseline (31.4±1.2) and this decrease was significant (*p*<0.001). No condition*time of day interaction effect was found (*p* = 0.86).

### Self-Reported Hunger, Appetite and Satiety

Subjects completed hunger, appetite and satiety visual analogue scales (VAS) on B1 and SR5. No changes were seen following sleep restriction in self reported hunger (*F*_1,87_ = 0.5, *p* = 0.49), appetite (*F*_1,87_ = 0.01, *p* = 0.92), or satiety (*F*_1,87_ = 0.2, *p* = 0.68), although a time of day effect was apparent for all variables (hunger (*F*_3,87_ = 19.9, *p*<0.001); appetite (*F*_3,87_ = 14.9, *p*<0.001); satiety (*F*_3,87_ = 20.9, *p*<0.001)).

## Discussion

Previous studies have shown impaired glucose metabolism following sleep restriction, a finding which is reflected in elevated glucose and insulin in the present study. This is possibly due to a rise in afternoon cortisol reflecting adrenal axis activation. Leptin levels also increased in an environment of adequate energy intake and reduced energy expenditure, but this increase did not lead to changes in appetite or hunger. This is in contrast to other published findings in the field which have previously documented decreases in leptin and increases in hunger and appetite following sleep restriction [17], [19]. A key difference between these studies and the present study was the inclusion of strict caloric control across the entire study duration which suggests that in the context of appropriate caloric intake, leptin levels are not significantly affected adversely by sleep restriction.

This is one of the first studies to examine the direct effect of sleep restriction on testosterone and sex hormone binding globulin (SHBG), which have both been linked in epidemiological findings with metabolic syndrome and insulin resistance. While testosterone did not change significantly with sleep restriction, a trend was observed, with levels reduced on SR5 compared to B1. SHBG decreased following sleep restriction, possibly due to increases in insulin which down-regulates SHBG [52]. These data have implications for healthy metabolic functioning.

It has been hypothesised that sleep restriction may elicit a physiological stress response that activates the hypothalamo-pituitary-adrenal (HPA) axis, increasing circulating cortisol. Chronically high levels of cortisol can impair insulin sensitivity and disrupt glucose metabolism, increasing the risk for type 2 diabetes. While a number of studies have found that sleep restriction does not increase overall mean cortisol [15], [16], [21], [45], [53], several others have found elevated cortisol in the latter part of the day instead of the usual diurnal decline [17], [18], [54]. Raised cortisol concentrations in the evening are thought to contribute to age-related insulin resistance [55], [56]. This is consistent with the current study which found that cortisol was elevated in the afternoon and evening and supports the hypothesis that sleep restriction may mimic pathophysiological processes that occur with ageing and lead to chronic age-related diseases such as type 2 diabetes and cardiovascular disease [18]. ACTH did not change with sleep restriction in the current study, but the levels were significantly decreased in the afternoon at 16:00, in a similar pattern to cortisol. These findings are unlikely to be a consequence of phase shift in circadian rhythms, as no observable shifts in the peak or trough of hormonal measures were evident following sleep restriction.

Consistent with previous research [15], [18]–[20], this study found that glucose tolerance to meals was impaired and insulin increased following sleep restriction suggesting that it may induce an insulin resistant state in healthy men. However, HOMA insulin resistance was not significantly different between B1 and SR5, suggesting that hepatic insulin sensitivity was not influenced by sleep restriction, although subtle changes in insulin resistance would not be detected by the HOMA method. The significant condition*time of day interaction in HOMA calculations suggest, but do not prove, potential postprandial effects. Such calculations when used after an oral glucose tolerance test form the basis for the Matsuda insulin resistance index, which is known to reflect peripheral, rather than hepatic, insulin sensitivity [57]. Our data suggest that peripheral rather than hepatic insulin resistance is induced with sleep restriction. If true, impaired peripheral insulin resistance in the presence of raised glucose as reported here would only occur with concomitant reduction in pancreatic beta cell function. Such a change is plausible [58]–[61], however we did not directly assess pancreatic beta cell function.

The finding of increased insulin following sleep restriction in the present study is in contrast to some previous findings. Of the previous studies that did not find changes in insulin [18], [19], [23], one allowed subjects to leave the laboratory during the day, thereby reducing control over snacking and naps [18], while another study used glucose tolerance tests [23]. These tests involve fasting and consumption of a glucose bolus which may impact glucose metabolism differently to the more naturalistic approach used in the current study where the focus was on the glucose and insulin response to meals and general changes in these outcomes after sleep restriction. Interestingly, no difference was observed between baseline and sleep restricted fasting glucose or insulin which, when also taking into consideration the marked response to meals, may indicate that sleep restriction impacts hepatic versus muscle glucose metabolism differentially. Further study is needed to examine this possibility.

The current study also utilized continuous glucose monitoring, which frequently sampled glucose for 24 h in the interstitial fluid at baseline and then after sleep restriction. This technology is unique as it is unobtrusive and enables long term monitoring of glucose with low burden to the study participant. Interstitial glucose followed a similar pattern to blood glucose in response to sleep restriction, with elevated interstitial glucose levels after meals and during the night-time period. The importance of this finding is twofold – it suggests that interstitial monitoring provides an accurate reflection of glucose levels from venous samples, and indicates the importance of continuous monitoring over nocturnal periods to truly unveil the effect of sleep restriction on glucose. This technology can be tolerated for extended periods of time and may be a useful tool in future studies to evaluate the cumulative effects of sleep restriction and/or displacement on glucose.

This study provided novel insight into the relationship between sleep restriction and SHBG, the glycoprotein predominantly responsible for the transport of testosterone. Following sleep restriction, SHBG levels were significantly reduced. This is particularly relevant to the relationship between sleep restriction and glucose metabolism, as low levels of endogenous SHBG have been linked with poor glucose tolerance [62], [63], and conversely, higher levels associated with reduced risk of type 2 diabetes [63], [64].

To our knowledge this was also the first study to examine the impact of sleep restriction on triglycerides in men. While cohort data have suggested that shorter sleep duration is associated with higher triglyceride levels [24], no experimental research has examined the impact of sleep restriction on triglycerides in men after more than two nights of sleep restriction. There have been some experimental investigations of FFA. These studies found that fasting FFA were unchanged after one night of sleep restriction [15], and significantly increased after two nights of sleep restriction [16] to 4 h TIB. In the current study triglycerides decreased following sleep restriction. This decrease could be due, in part, to differences in the study subjects' diet prior to the laboratory phase of the study. As diet was not monitored prior to the laboratory phase of the study (with the exception of alcohol and caffeine consumption, which was not allowed), it is not known if subjects routinely ate more fats and carbohydrates than they consumed during the laboratory phase of study. Triglycerides are known to increase with a carbohydrate rich diet, and also decrease when dietary intake of refined carbohydrates is reduced [65]. The macronutrients of the diet during the laboratory phase of the study were balanced and subjects were provided with a range of foods including salads and quality protein. A rapid change in their diet, including a reduction in the amount of carbohydrates consumed, relative to habitual food intake could have produced the small decrease in triglycerides observed in the present study. As such, the change in triglycerides may not represent a change that would occur outside the laboratory environment.

Early experimental studies exploring the relationship between sleep restriction and the adipose-derived hormone leptin found decreases after both two [19] and six [17] nights of sleep restriction to 4 h TIB. In one study, these decreases were aligned with self reported increases in hunger and appetite [19]. This prompted the hypothesis that sleep restriction is linked with obesity via disruption of leptin and increased cravings for carbohydrate rich foods [17]. However energy intake was restricted in these studies which may have resulted in an increased leptin response to the restricted calories rather than a direct effect of sleep restriction on leptin [35]. In more recent investigations, in which subjects were either allowed BMI-appropriate food intake or ad libitum food access, leptin was unaffected [30], [32], [66] or elevated [20], [29], [31].

The current study found a significant increase in leptin following sleep restriction in line with these and recent cohort [67] studies. In the present study, food was controlled to an appropriate caloric intake for subjects' weight, height, age and activity level and subjects did not report significant changes following sleep restriction in hunger, appetite and satiety. Time of day effects apparent in this self-report data are unsurprising given the timing of test bouts pre lunch and post dinner. The leptin increase seen in our data was likely due to the adequate energy intake and the sedentary lifestyle subjects experienced in the laboratory, suggesting little change in overall energy balance. However, if calories are restricted during sleep loss it seems that leptin will decrease to help correct the energy imbalance and protect against changes in nutrient supply. The link between sleep loss and obesity therefore appears more complicated than a simple disruption of leptin metabolism.

The effect of sleep restriction on testosterone has received little attention, despite the fact that the majority of experimental sleep restriction and metabolic studies have been conducted in men. Research on the relationship between sleep and testosterone has predominantly focused on the effect of total sleep deprivation [43], [44], [68], or examined the differential effects of sleep stages on testosterone [69]–[73]. Given the relationship between total sleep time and morning testosterone [72], it is unsurprising that the only published data to date on the relationship between sleep restriction and testosterone found that levels decreased significantly after 8 nights of 5 hours time in bed [45]. While testosterone did not decrease following sleep restriction in the current study, there was a clear trend, consistent with recent findings [45]. The timing and the duration of the sleep restriction period differed considerably between the two studies (8 nights of 5 h TIB, 12:30–05:30 [45] versus 5 nights of 4 h TIB, 04:00–08:00) and it is possible, because of the relationship that testosterone has with total sleep time as well as its circadian variability, that greater periods of sleep loss or loss of certain sleep stages, such as REM sleep, are needed to observe an effect. These results are especially important for older men who regularly restrict their sleep. Testosterone decreases with age and the further insult of chronically reduced sleep could have important implications for health status, given the role of testosterone in a number of physical and psychological conditions such as sexual function [74], muscle and bone mass [75], and mood [76].

While this study was limited to male participants, a low SHBG has also been shown to be associated with obesity [77], [78], insulin resistance [78], [79], and the metabolic syndrome in women [80]. SHBG is a marker of de novo hepatic lipogenesis in both men and women [81], [82]. The apparent contradiction that low testosterone levels are associated with insulin resistance and the metabolic syndrome in men when the association is with high testosterone levels in women is the result of the decrease in SHBG and its consequent effect on the assessment of testosterone in each sex [81].

The results of this study should be considered in light of several methodological constraints. After the fasting blood draw, blood sampling occurred at approximately 2 hourly intervals across B1 and SR5 and did not include nocturnal sampling. This means that fluctuations in between the blood draws were not captured, and makes it difficult to compare with studies that sampled blood more frequently. Further, it does not enable comment on nocturnal patterns of hormonal levels. This study was also only able to sample blood before and after sleep restriction at one particular dose, and so the dose-response (sleep restriction dose and number of days of exposure) aspects of the effects of sleep restriction on metabolic and hormonal measures cannot be determined.

Finally, as with the majority of laboratory-based studies of sleep restriction and metabolic regulation, this study only investigated healthy, young adult, male subjects, and findings may not generalize to women, to other age categories, and to clinical populations. Nevertheless, this study is the first to examine the effect of sleep restriction on glucose, insulin, triglycerides and potentially underlying hormonal mechanisms in concert. Our findings indicate that short term sleep curtailment leads to changes in glucose metabolism and adrenal reactivity. When experienced chronically, these changes may increase the risk for type 2 diabetes.

## References

[1] Reynolds AC, Banks S, Kerkhof GA, Van Dongen HPA (2010). Total sleep deprivation, chronic sleep restriction and sleep disruption..

[2] Tasali E, Leproult R, Spiegel K (2009). Reduced sleep duration or quality: Relationships with insulin resistance and type 2 diabetes.. Prog Cardiovasc Dis.

[3] Magee CA, Iverson DC, Caputi P (2010). Sleep duration and obesity in middle-aged Australian adults.. Obesity (Silver Spring).

[4] Vgontzas AN, Bixler EO (2008). Short sleep and obesity: Are poor sleep, chronic stress, and unhealthy behaviors the link?. Sleep.

[5] Cappuccio FP, Taggart FM, Kandala NB, Currie A, Peile E (2008). Meta-analysis of short sleep duration and obesity in children and adults.. Sleep.

[6] Jennings JR, Muldoon MF, Hall M, Buysse DJ, Manuck SB (2007). Self-reported sleep quality is associated with the metabolic syndrome.. Sleep.

[7] Chaput JP, Despres JP, Bouchard C, Tremblay A (2007). Association of sleep duration with type 2 diabetes and impaired glucose tolerance.. Diabetologia.

[8] Knutson KL, Ryden AM, Mander BA, Van Cauter E (2006). Role of sleep duration and quality in the risk and severity of type 2 diabetes mellitus.. Arch Int Med.

[9] Yaggi HK, Araujo AB, McKinlay JB (2006). Sleep duration as a risk factor for the development of type 2 diabetes.. Diabetes Care.

[10] (2002). Third Report of the National Cholesterol Education Program (NCEP) Expert Panel on Detection, Evaluation, and Treatment of High Blood Cholesterol in Adults (Adult Treatment Panel III) final report.. Circulation.

[11] Balkau B, Charles MA (1999). Comment on the provisional report from the WHO consultation. European Group for the Study of Insulin Resistance (EGIR).. Diabetic Medicine.

[12] Einhorn D, Reaven GM, Cobin RH, Ford E, Ganda OP (2003). American College of Endocrinology position statement on the insulin resistance syndrome.. Endocrine Practice.

[13] Alberti KG, Zimmet P, Shaw J (2006). Metabolic syndrome-a new world-wide definition. A Consensus Statement from the International Diabetes Federation.. Diabetic Medicine.

[14] WHO (1999). Definition and Classification of Diabetes Mellitus and its Complications Report of a WHO Consultation. Part 1: Diagnosis and Classification of Diabetes Mellitus.. World Health Organisation, Geneva.

[15] Donga E, van Dijk M, van Dijk JG, Biermasz NR, Lammers GJ (2010). A single night of partial sleep deprivation induces insulin resistance in multiple metabolic pathways in healthy subjects.. J Clin Endocrinol Metab.

[16] Schmid SM, Hallschmid M, Jauch-Chara K, Wilms B, Lehnert H (2011). Disturbed glucoregulatory response to food intake after moderate sleep restriction.. Sleep.

[17] Spiegel K, Leproult R, L'Hermite-Baleriaux M, Copinschi G, Penev PD (2004). Leptin levels are dependent on sleep duration: Relationships with sympathovagal balance, carbohydrate regulation, cortisol, and thyrotropin.. J Clin Endocrinol Metab.

[18] Spiegel K, Leproult R, Van Cauter E (1999). Impact of sleep debt on metabolic and endocrine function.. Lancet.

[19] Spiegel K, Tasali E, Penev P, Van Cauter E (2004). Brief communication: Sleep curtailment in healthy young men is associated with decreased leptin levels, elevated ghrelin levels, and increased hunger and appetite.. Ann Intern Med.

[20] van Leeuwen WM, Hublin C, Sallinen M, Harma M, Hirvonen A (2010). Prolonged sleep restriction affects glucose metabolism in healthy young men.. Int J Endocrinol.

[21] Vgontzas AN, Zoumakis E, Bixler EO, Lin HM, Follett H (2004). Adverse effects of modest sleep restriction on sleepiness, performance, and inflammatory cytokines.. J Clin Endocrinol Metab.

[22] Spiegel K, Knutson K, Leproult R, Tasali E, Van Cauter E (2005). Sleep loss: A novel risk factor for insulin resistance and Type 2 diabetes.. J Appl Physiol.

[23] Nedeltcheva AV, Kessler L, Imperial J, Penev PD (2009). Exposure to recurrent sleep restriction in the setting of high caloric intake and physical inactivity results in increased insulin resistance and reduced glucose tolerance.. J Clin Endocrinol Metab.

[24] Bjorvatn B, Sagen IM, Oyane N, Waage S, Fetveit A (2007). The association between sleep duration, body mass index and metabolic measures in the Hordaland Health Study.. J Sleep Res.

[25] Kerkhofs M, Boudjeltia KZ, Stenuit P, Brohee D, Cauchie P (2007). Sleep restriction increases blood neutrophils, total cholesterol and low density lipoprotein cholesterol in postmenopausal women: A preliminary study.. Maturitas.

[26] Khani S, Tayek JA (2001). Cortisol increases gluconeogenesis in humans: Its role in the metabolic syndrome.. Clinical Science.

[27] Wu H, Zhao Z, Stone WS, Huang L, Zhuang J (2008). Effects of sleep restriction periods on serum cortisol levels in healthy men.. Brain Res Bull.

[28] Mantzoros CS, Magkos F, Brinkoetter M, Sienkiewicz E, Dardeno TA (2011). Leptin in human physiology and pathophysiology.. Am J Physiol Endocrinol and Metab.

[29] Simpson NS, Banks S, Dinges DF (2010). Sleep Restriction Is Associated With Increased Morning Plasma Leptin Concentrations, Especially in Women.. Biol Res Nurs.

[30] Schmid SM, Hallschmid M, Jauch-Chara K, Wilms B, Benedict C (2009). Short-term sleep loss decreases physical activity under free-living conditions but does not increase food intake under time-deprived laboratory conditions in healthy men.. Am J Clin Nutr.

[31] Omisade A, Buxton OM, Rusak B (2010). Impact of acute sleep restriction on cortisol and leptin levels in young women.. Physiol Behav.

[32] Nedeltcheva AV, Kilkus JM, Imperial J, Kasza K, Schoeller DA (2009). Sleep curtailment is accompanied by increased intake of calories from snacks.. Am J Clin Nutr.

[33] Mullington JM, Chan JL, Van Dongen HP, Szuba MP, Samaras J (2003). Sleep loss reduces diurnal rhythm amplitude of leptin in healthy men.. J Neuroendocrinol.

[34] Schmid SM, Hallschmid M, Jauch-Chara K, Born J, Schultes B (2008). A single night of sleep deprivation increases ghrelin levels and feelings of hunger in normal-weight healthy men.. J Sleep Res.

[35] Penev PD (2011). Short sleep and circulating adipokine concentrations: Does the fat hit the fire?. Sleep.

[36] Mauras N, Hayes V, Welch S, Rini A, Helgeson K (1998). Testosterone deficiency in young men: Marked alterations in whole body protein kinetics, strength, and adiposity.. J Clin Endocrinol Metab.

[37] Seidell JC, Bjorntorp P, Sjostrom L, Kvist H, Sannerstedt R (1990). Visceral fat accumulation in men is positively associated with insulin, glucose, and C-peptide levels, but negatively with testosterone levels.. Metabolism.

[38] Katabami T, Kato H, Asahina T, Hinohara S, Shin T (2010). Serum free testosterone and metabolic syndrome in Japanese men.. Endocr J.

[39] Zitzmann M (2009). Testosterone deficiency, insulin resistance and the metabolic syndrome.. Nat Rev Endocrinol.

[40] Li C, Ford ES, Li B, Giles WH, Liu S (2010). Association of testosterone and sex hormone-binding globulin with metabolic syndrome and insulin resistance in men.. Diabetes Care.

[41] Stellato RK, Feldman HA, Hamdy O, Horton ES, McKinlay JB (2000). Testosterone, sex hormone-binding globulin, and the development of type 2 diabetes in middle-aged men: prospective results from the Massachusetts male aging study.. Diabetes Care.

[42] Birkeland KI, Hanssen KF, Torjesen PA, Vaaler S (1993). Level of sex hormone-binding globulin is positively correlated with insulin sensitivity in men with type 2 diabetes.. J Clin Endocrinol Metab.

[43] Akerstedt T, Palmblad J, de la Torre B, Marana R, Gillberg M (1980). Adrenocortical and gonadal steroids during sleep deprivation.. Sleep.

[44] Cortes-Gallegos V, Castaneda G, Alonso R, Sojo I, Carranco A (1983). Sleep deprivation reduces circulating androgens in healthy men.. Arch Androl.

[45] Leproult R, Van Cauter E (2011). Effect of 1 week of sleep restriction on testosterone levels in young healthy men.. JAMA.

[46] Anderson DC (1974). Sex-hormone-binding globulin.. Clin Endocrinol.

[47] Smith CS, Reilly C, Midkiff K (1989). Evaluation of three circadian rhythm questionnaires with suggestions for an improved measure of morningness.. J Appl Psychol.

[48] Buysse DJ, Reynolds CF, 3rd, Monk TH, Berman SR, Kupfer DJ (1989). The Pittsburgh Sleep Quality Index: A new instrument for psychiatric practice and research.. Psychiatry Research.

[49] Keenan DB, Mastrototaro JJ, Voskanyan G, Steil GM (2009). Delays in minimally invasive continuous glucose monitoring devices: A review of current technology.. J Diabetes Sci Technol.

[50] Kaufman FR, Gibson LC, Halvorson M, Carpenter S, Fisher LK (2001). A pilot study of the continuous glucose monitoring system: Clinical decisions and glycemic control after its use in pediatric type 1 diabetic subjects.. Diabetes Care.

[51] Bode BW, Gross TM, Thornton KR, Mastrototaro JJ (1999). Continuous glucose monitoring used to adjust diabetes therapy improves glycosylated hemoglobin: A pilot study.. Diabetes Res Clin Pract.

[52] Nestler JE (1993). Sex hormone-binding globulin: A marker for hyperinsulinemia and/or insulin resistance?. J Clin Endocrinol Metabl.

[53] Schmid SM, Jauch-Chara K, Hallschmid M, Schultes B (2009). Mild sleep restriction acutely reduces plasma glucagon levels in healthy men.. J Clin Endocrinol Metab.

[54] Buxton OM, Pavlova M, Reid EW, Wang W, Simonson DC (2010). Sleep restriction for 1 week reduces insulin sensitivity in healthy men.. Diabetes.

[55] Dallman MF, Strack AM, Akana SF, Bradbury MJ, Hanson ES (1993). Feast and famine: Critical role of glucocorticoids with insulin in daily energy flow.. Front Neuroendocrinol.

[56] Kern W, Dodt C, Born J, Fehm HL (1996). Changes in cortisol and growth hormone secretion during nocturnal sleep in the course of aging.. J Gerontol A Biol Sci Med Sci.

[57] Matsuda M (2010). Measuring and estimating insulin resistance in clinical and research settings.. Nutrition, Metabolism, and Cardiovascular Diseases.

[58] Gale JE, Cox HI, Qian J, Block GD, Colwell CS (2011). Disruption of circadian rhythms accelerates development of diabetes through pancreatic beta-cell loss and dysfunction.. Journal of Biological Rhythms.

[59] Ban HJ, Kim SC, Seo J, Kang HB, Choi JK (2011). Genetic and metabolic characterization of insomnia.. PloS one.

[60] Marcheva B, Ramsey KM, Buhr ED, Kobayashi Y, Su H (2010). Disruption of the clock components CLOCK and BMAL1 leads to hypoinsulinaemia and diabetes.. Nature.

[61] Sadacca LA, Lamia KA, deLemos AS, Blum B, Weitz CJ (2011). An intrinsic circadian clock of the pancreas is required for normal insulin release and glucose homeostasis in mice.. Diabetologia.

[62] Lindstedt G, Lundberg PA, Lapidus L, Lundgren H, Bengtsson C (1991). Low sex-hormone-binding globulin concentration as independent risk factor for development of NIDDM. 12-yr follow-up of population study of women in Gothenburg, Sweden.. Diabetes.

[63] Ding EL, Song Y, Malik VS, Liu S (2006). Sex differences of endogenous sex hormones and risk of type 2 diabetes: a systematic review and meta-analysis.. JAMA.

[64] Perry JR, Weedon MN, Langenberg C, Jackson AU, Lyssenko V (2010). Genetic evidence that raised sex hormone binding globulin (SHBG) levels reduce the risk of type 2 diabetes.. Hum Mol Genet.

[65] O'Keefe JH, Gheewala NM, O'Keefe JO (2008). Dietary strategies for improving post-prandial glucose, lipids, inflammation, and cardiovascular health.. J Am Coll Cardiol.

[66] Nedeltcheva AV, Kilkus JM, Imperial J, Schoeller DA, Penev PD (2010). Insufficient sleep undermines dietary efforts to reduce adiposity.. Ann Int Med.

[67] Hayes AL, Xu F, Babineau D, Patel SR (2011). Sleep duration and circulating adipokine levels.. Sleep.

[68] Gonzalez-Santos MR, Gaja-Rodriguez OV, Alonso-Uriarte R, Sojo-Aranda I, Cortes-Gallegos V (1989). Sleep deprivation and adaptive hormonal responses of healthy men.. Arch Androl.

[69] Dray F, Reinberg A, Sebaoun J (1965). Biological rhythm of plasma free testosterone in healthy adult males: existence of a circadian variation.. CR Acad Sci Hebd Seances Acad Sci D.

[70] Resko JA, Eik-nes KB (1966). Diurnal testosterone levels in peripheral plasma of human male subjects.. J Clin Endocrinol Metab.

[71] Penev P, Spiegel K, L'Hermite-Baleriaux M, Schneider R, Van Cauter E (2003). Relationship between REM sleep and testosterone secretion in older men.. Ann Endocrinol.

[72] Penev PD (2007). Association between sleep and morning testosterone levels in older men.. Sleep.

[73] Luboshitzky R, Herer P, Levi M, Shen-Orr Z, Lavie P (1999). Relationship between rapid eye movement sleep and testosterone secretion in normal men.. Journal of Andrology.

[74] Wang C, Jackson G, Jones TH, Matsumoto AM, Nehra A (2011). Low testosterone associated with obesity and the metabolic syndrome contributes to sexual dysfunction and cardiovascular disease risk in men with type 2 diabetes.. Diabetes Care.

[75] Lakshman KM, Araujo AB, Bhasin S, Orwoll ES, Bilezikian JP, Vanderschueren D (2010). Testosterone therapy for osteoporosis in men..

[76] Joshi D, van Schoor NM, de Ronde W, Schaap LA, Comijs HC (2010). Low free testosterone levels are associated with prevalence and incidence of depressive symptoms in older men.. Clin Endocrinol.

[77] Sherif K, Kushner H, Falkner BE (1998). Sex hormone-binding globulin and insulin resistance in African-American women.. Metabolism: Clinical and Experimental.

[78] Akin F, Bastemir M, Alkis E, Kaptanoglu B (2008). Associations between sex hormone binding globulin and metabolic syndrome parameters in premenopausal obese women.. Indian Journal of Medical Sciences.

[79] Preziosi P, Barrett-Connor E, Papoz L, Roger M, Saint-Paul M (1993). Interrelation between plasma sex hormone-binding globulin and plasma insulin in healthy adult women: The telecom study.. J Clin Endocrinol Metab.

[80] Brand JS, van der Tweel I, Grobbee DE, Emmelot-Vonk MH, van der Schouw YT (2011). Testosterone, sex hormone-binding globulin and the metabolic syndrome: A systematic review and meta-analysis of observational studies.. International Journal of Epidemiology.

[81] Hammond GL, Wu TS, Simard M (2012). Evolving utility of sex hormone-binding globulin measurements in clinical medicine.. Current Opinion in Endocrinology, Diabetes, and Obesity.

[82] Hammond GL (2011). Diverse roles for sex hormone-binding globulin in reproduction.. Biology of Reproduction.

